# A 3-year follow-up study of radiotherapy using computed tomography–based image-guided brachytherapy for cervical cancer

**DOI:** 10.1093/jrr/rry104

**Published:** 2019-01-11

**Authors:** Atsushi Kawashima, Fumiaki Isohashi, Seiji Mabuchi, Kenjiro Sawada, Yutaka Ueda, Eiji Kobayashi, Yuri Matsumoto, Keisuke Otani, Keisuke Tamari, Yuji Seo, Osamu Suzuki, Iori Sumida, Takuji Tomimatsu, Tadashi Kimura, Kazuhiko Ogawa

**Affiliations:** 1 Department of Radiation Oncology, Osaka University Graduate School of Medicine, 2-2 (D10) Yamada-oka, Suita, Osaka, Japan; 2 Department of Obstetrics and Gynecology, Osaka University Graduate School of Medicine, 2-2 (D10) Yamada-oka, Suita, Osaka, Japan

**Keywords:** cervical cancer, image-guided brachytherapy, computed tomography, three-dimensional treatment planning

## Abstract

Outcomes for patients with Stage IB1–IVA cervical cancer treated with computed tomography (CT)-based image-guided brachytherapy (IGBT) were examined in this study. A total of 84 patients were analyzed between March 2012 and June 2015. Whole-pelvic radiotherapy with a central shield was performed for each patient, and the total pelvic sidewall dose was 50 Gy. IGBT was delivered in 2–4 fractions. The initial prescription dose (6.8 Gy) was delivered at Point A, and the dose distribution was modified manually by graphical optimization. The total dose was calculated as the biologically equivalent dose in 2 Gy fractions (EQD2). Concurrent chemotherapy was administered to 64 patients (76%). The median follow-up period was 36 months (range 2–62 months). The 3-year overall survival, local control, and progression-free survival rates were 94%, 89% and 81%, respectively. The mean EQD2 for HR-CTV D_90_ was 73.4 Gy, and the EQD2 for HR-CTV D_90_ was not significantly associated with the local control rate. In multivariate analysis, adenocarcinoma (*P* = 0.03) and tumor size ≥45 mm (*P* = 0.06) were risk factors for local control. The patients were divided into four groups based on histology (squamous cell carcinoma vs adenocarcinoma) and tumor size (<45 vs ≥45 mm). Those with large adenocarcinomas had significantly worse outcomes. In conclusion, CT-based IGBT achieved favorable local control, but different treatment strategies may be necessary for large adenocarcinomas.

## INTRODUCTION

Brachytherapy has played the most important role in the treatment of cervical cancer to date [1]. Brachytherapy combined with external beam radiotherapy (EBRT) has been established as a standard radical treatment in patients with cervical cancer. Treatment planning for brachytherapy has changed from the use of conventional 2D X-rays with a dose prescription (to standard points) to 3D image–guided volume-based brachytherapy (3D-IGBT). Since the Groupe Européen de Curietherapie/European Society for Therapeutic Radiology and Oncology (GEC-ESTRO) working group recommendations were published in 2005 [2], magnetic resonance imaging (MRI)-based planning has been viewed as the gold standard for cervical cancer brachytherapy, and several studies have reported excellent local control (LC) and few complications with MRI-based planning [3, 4]. However, many centers, including many of those in Japan, utilize computed tomography (CT) instead of MRI for brachytherapy planning. This is because of the cost of MRI, the need for dedicated devices, and the lack of accessibility for the radiotherapy (RT) department, and there are several reports of studies of CT-based brachytherapy planning [5–9]. We have used CT-based 3D-IGBT in all brachytherapy sessions since March 2012. The purpose of this study was to analyze our 3-year follow-up results for cervical cancer patients treated with CT-based 3D-IGBT.

## MATERIALS AND METHODS

### Patients

This study was performed as a retrospective chart review and was approved by our institutional review board. A total of 99 patients with cervical cancer were treated at our hospital with high-dose-rate (HDR) CT-based IGBT between March 2012 and June 2015. Fifteen patients were excluded due to para-aortic lymph node metastasis (*n* = 5), a rare histology with carcinosarcoma (*n* = 1), performance of template-based interstitial brachytherapy (ISBT) (*n* = 6), and loss from follow-up (*n* = 3). Patients with lower para-aortic node metastasis (below the level of the inferior mesenteric artery) were included, because the whole-pelvic field contained the lesion. Thus, data from 84 patients were analyzed.

### External beam radiotherapy

EBRT was delivered by CT-based treatment planning at a dose of 2 Gy per fraction. The clinical target volume (CTV) included the gross tumor volume (GTV), cervix, parametria, uterus, upper part of the vagina, and regional lymph nodes (common, presacral, and internal and external iliac). The dose of the whole-pelvic RT (WPRT) before central shielding (CS) depended on the initial size of the tumor: patients with tumors <4 cm and ≥4 cm in diameter received 30 Gy and 40 Gy, respectively, as the minimum dose. The initial 30–40 Gy was delivered by WPRT with a 4-field box, and pelvic irradiation was then delivered with a 4 cm-wide CS. The total pelvic side wall dose was 50 Gy. To monitor tumor shrinkage, patients received a weekly pelvic examination and transvaginal or transrectal ultrasound by gynecological and radiation oncologists. If it was unlikely that the whole tumor volume could be irradiated by intracavitary brachytherapy (ICBT) after 40 Gy, an additional WP dose of 10 Gy without CS was delivered. For patients with pelvic lymph nodes of diameter >25 mm, an additional boost of 6–10 Gy was administered.

### Intracavitary brachytherapy

After adequate tumor regression, HDR-ICBT was performed once a week during and after the course of EBRT with CS. MRI just before the first session of brachytherapy was only performed in some patients during this study period. Four, three and two fractions of ICBT were administered to patients who received WPRT at 30, 40 and 50 Gy, respectively. Patients with a large or complex tumor who required template-based ISBT were excluded from the study [10]. ICBT was administered using a microSelectron digital (HDR-V3) brachytherapy afterloader (Elekta Inc., Stockholm, Sweden) with Fletcher-type (Fletcher-Williamson Asia Pacific) metal applicators (Elekta Inc.), each of which is comprised of one curved central tandem applicator and two non-shielded ovoid applicators. For patients with vaginal infiltration or a narrow vagina, the tandem with the vaginal cylinder was placed to fit the cavity of uterine body and vagina. No patients were treated with a combination of ICBT applicators and interstitial needles. A planning CT scan was obtained before the delivery of each fraction. The high-risk CTV (HR-CTV) and organs at risk (OARs) were contoured on the planning CT with Oncentra® (Elekta Inc.), based on several guidelines [11, 12]. Initially, the ICBT dose was prescribed at Point A, which was defined as 2 cm above the cervical os marker and 2 cm perpendicularly lateral to the tandem. The initial planned dose of HDR-ICBT was 6.8 Gy. After standard loading of the source dwell positions and weighting with the fraction dose at Point A, the dwell times were modified manually by graphical optimization to maximize the coverage of the HR-CTV while reducing the dose to the OARs to meet our dose constraints: HR-CTV D_90_ > 6.0 Gy, rectum D_2cm^3^_ < 7.0 Gy, and bladder D_2cm^3^_ < 7.0 Gy. EBRT was omitted on the days when HDR-ICBT was performed. The biologically equivalent dose in 2-Gy fractions (EQD2) was calculated as the combined dose of ICBT and EBRT (excluding the fractions with CS). A value of α/β = 10 was assumed for tumors, and a value of α/β = 3 was assumed for OARs.

### Chemotherapy

Concurrent chemotherapy was administered to 64 patients (76%), including weekly nedaplatin (35 mg/m^2^) in 39 patients, weekly cisplatin (40 mg/m^2^) in 8 patients, and weekly carboplatin (area under the curve: 2.0, Calvert’s formula) plus paclitaxel (35 mg/m^2^) in 17 patients [13, 14]. Patients aged 75 years or older, who had either renal failure or an allergy to platinum agents, were treated without chemotherapy.

### Follow-up

Patients were followed-up by gynecological and radiation oncologists, as described elsewhere [15]. Complications were assessed using the Common Terminology Criteria for Adverse Events v. 4.0.

### Statistical analysis

Overall survival (OS), progression-free survival (PFS), and LC were determined from the beginning of RT using the Kaplan–Meier method. Differences between factors were examined by the log-rank test. Relationships between patient-related factors and treatment factors were analyzed by the Fisher exact test. Receiver operating characteristics (ROC) curve analysis was performed to select the most relevant threshold. The mean dose–volume histogram (DVH) parameters for HR-CTV D_90_ and D_100_ with or without local recurrence, and the mean DVH parameters per application before or after graphical optimization were compared by the Mann–Whitney U test. The predictive value of the tumor size and histology for local recurrence was evaluated with a binary logistic regression model. Development of local recurrence was used as the quantal endpoint. All statistical analyses were performed with R Ver. 3.4.3. (the R Foundation for Statistical Computing, Vienna, Austria). A *P*-value of <0.05 or a 95% confidence interval not encompassing 1.0 were considered to indicate significant difference. All statistical tests were two-sided.

## RESULTS

**Fig. 1.**
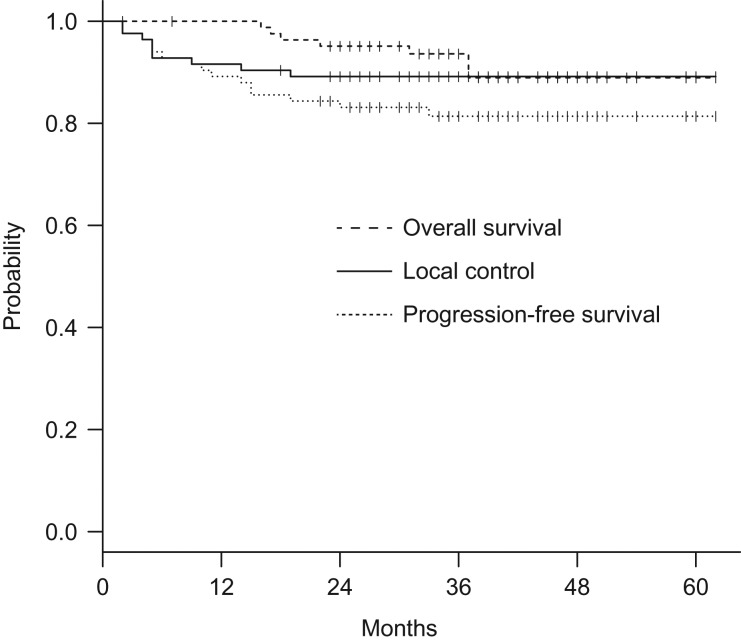
Kaplan–Meier estimates of overall survival (red line), progression-free survival (green line) and local control (black line) of all patients (*n* = 84).

The characteristics of the patients are shown in Table 1. The median follow-up period was 36 months (range 2–62 months) and the median age at diagnosis was 61 years (range 30–93 years). Sixteen patients (19%) developed recurrent lesions: local recurrence in 9 patients, regional recurrence in 9 patients, and distant recurrence in 14 patients, with some patients having multiple events. The 3-year OS, LC and PFS rates were 94%, 89% and 81%, respectively (Fig. 1).

**Table 1.** Patient characteristics (*n* = 84)

Characteristics	*n*	%
Age (years)		
Median (range)	61 (30–93)	
FIGO		
IB1	24	28
IB2	3	4
IIA1	4	5
IIA2	5	6
IIB	36	43
IIIA	1	1
IIIB	10	12
IVA	1	1
Pelvic LN		
Positive	28	33
Negative	56	67
Pathology		
SCC	71	85
AD	13	15
Tumor size (mm)		
Median (range)	38 (8–72)	
Chemotherapy		
with	64	76
without	20	24

FIGO = International Federation of Gynecology and Obstetrics, LN = lymph node, SCC = squamous cell carcinoma, AD = adenocarcinoma.

The DVH parameters are summarized in Table 2. The mean EQD2 for HR-CTV D_90_, HR-CTV D_100_, rectum D_2cm^3^_ and bladder D_2cm^3^_ were 73.4 Gy, 58.8 Gy, 67.1 Gy and 63.6 Gy, respectively. Graphical optimization was performed in 99 applications delivered to 30 patients (36%). The purpose of optimization was to improve the coverage of HR-CTV in 33 patients (33%) and to reduce the dose to the OARs in 66 patients (67%). In patients with improvement in the HR-CTV group, there were increases in the HR-CTV D_90_ (6.11 ± 0.68 to 6.70 ± 0.44 Gy), rectum D_2cm^3^_ (4.98 ± 1.01 to 5.33 ± 1.11 Gy) and bladder D_2cm^3^_ (4.97 ± 0.72 to 5.30 ± 0.76 Gy), but the rise in the D_2cm^3^_ for the OARs was within the limit of the dose constraints. In the group of patients with a reduction in the OARs, there were decreases in the HR-CTV D_90_ (8.24 ± 1.26 to 7.77 ± 1.14 Gy), rectum D_2cm^3^_ (5.24 ± 1.41 to 5.02 ± 1.25 Gy) and bladder D_2cm^3^_ (6.14 ± 1.26 to 5.74 ± 0.86 Gy), but the reduction in the HR-CTV D_90_ was at the upper limit of the dose constraints and was maintained at >6 Gy.

**Table 2.** Summary of dose–volume histogram parameters (Gy)

	Overall	LC+	LC−	*P*
HR-CTV D_90_	73.4 (±7.5)	73.5 (±7.5)	72.2 (±7.1)	0.53
HR-CTV D_100_	58.8 (±5.9)	58.8 (±6.0)	58.6 (±5.3)	0.81
Rectum D_2cm^3^_	67.1 (±7.5)			
Bladder D_2cm^3^_	63.6 (±8.7)			

LC = local control; HR-CTV = high-risk clinical target volume; D_90_, D_100_ and D_2cm^3^_ = minimum dose received by the 90%, 100% and 2 cm^3^ volumes with highest irradiation. Data are shown ± the standard deviation.

The results of the univariate analysis of the prognostic factors for LC are shown in Table 3. ROC curve analysis was used to determine each cut-off parameter. Histology [squamous cell carcinoma (SCC) vs adenocarcinoma (AD)], tumor size (<45 vs ≥45 mm) and HR-CTV in the first ICBT session (<35 vs ≥35 cm^3^) were significantly associated with LC. However, HR-CTV D_90_ and HR-CTV D_100_ did not show this relationship, and the mean values of HR-CTV D_90_ and HR-CTV D_100_ did not differ significantly between cases with and without local recurrence (Table 2). In multivariate analysis using the significant factors from the univariate analysis, histology emerged as a significant risk factor for LC (*P* = 0.03), and tumor size showed a tendency to be a risk factor for LC (*P* = 0.06) (Table 4).

**Table 3.** Univariate analysis for correlation of clinical factors with local control

	Failure(−)	Failure(+)	*P*
Chemotherapy			
with	55	9	0.106
without	20	0	
FIGO			
IB1-IIA2	34	2	0.289
IIB-IVA	41	7	
Histology			
SCC	66	5	0.029
AD	9	4	
Pelvic LN			
negative	52	4	0.152
positive	23	5	
Tumor size (mm)			
<45	48	1	0.003
≥45	27	8	
HR-CTV volume (cm^3^)			
<35	61	4	0.025
≥35	14	5	
HR-CTV D_90_ (Gy)			
<67	10	3	0.141
≥67	65	6	
HR-CTV D_100_ (Gy)			
<55	13	3	
≥55	62	6	0.363

FIGO = International Federation of Gynecology and Obstetrics, SCC = squamous cell carcinoma, AD = adenocarcinoma, LN = lymph node, HR-CTV = high-risk clinical target volume, D_90_ and D_100_ = minumum dose received by the 90% and 100% volumes with highest irradiation, respectively.

**Table 4.** Multivariate analysis for local control

	HR	95% CI	*P*
Histology (SCC vs AD)	5.25	1.17–23.68	0.03
Size (<45 vs ≥45 mm)	1.06	0.99–1.13	0.06
HR-CTV volume (<35 vs ≥35 cm^3^)	1.04	0.99–1.09	0.11

HR = hazard ratio, CI = confidence interval, SCC = squamous cell carcinoma, AD = adenocarcinoma, HR-CTV = high-risk clinical target volume.

**Fig. 2.**
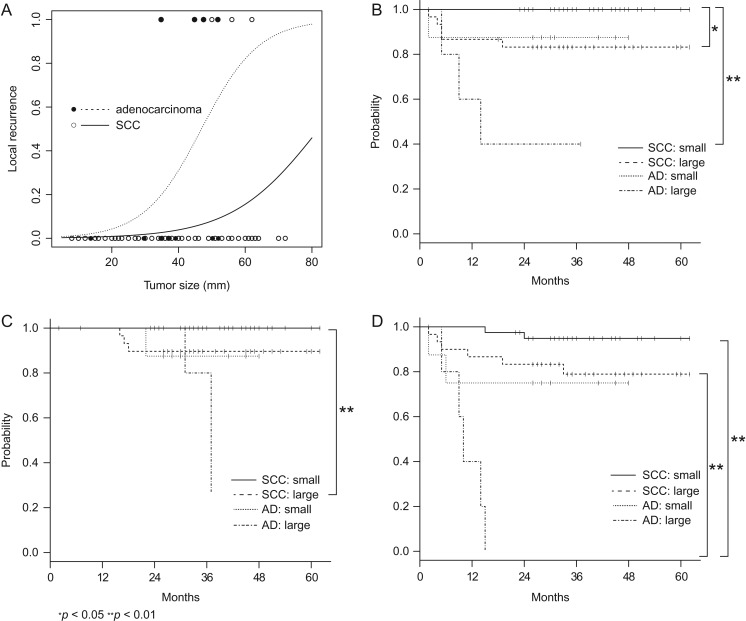
A binary logistic regression model of tumor size and histology for prediction of local recurrence (A). Kaplan–Meier estimates of local control (B), overall survival (C), and progression-free survival (D), stratified into four groups according to histology [squamous cell carcinoma (SCC) vs adenocarcinoma (AD)] and tumor size (<45 vs ≥45 mm).

Next, we investigated how histology and tumor size affected LC. The slope of the logistic regression curve in AD was steeper than that for SCC, which indicated that the relationship of local recurrence risk and tumor size had a larger impact in patients with AD than in those with SCC (Fig. 2A). The patients were also divided into four groups based on histology (SCC vs AD) and tumor size (<45 vs ≥45 mm). A log-rank test indicated that patients with a large AD had significantly worse LC and OS than those with a small SCC. In addition, patients with a large AD had worse PFS than those with a small or large SCC (Fig. 2B–D).

Four patients (5%) had gastrointestinal morbidity of Grade 3, including two with proctitis requiring endoscopic hemostasis and one with ileus requiring elective surgery. The D_2cm^3^_ doses to the rectum were 83.5 and 75.4 Gy to the two patients with proctitis, respectively, and 73.5 Gy to the one with ileus. At the 3-year follow-up after RT, one patient had severe ileal adhesion to the dorsal aspect of the uterine cervix, which caused perforation of the ileum. The D_2cm^3^_ to the ileum was 68.7 Gy. Twenty patients (24%) had Grade 1–2 proctitis. No patients had a urinary toxicity of Grade ≥2.

## DISCUSSION

Clinical outcomes and complications for patients with cervical cancer treated by CT-based 3D-IGBT with an intracavitary applicator were analyzed in this study. The 3-year OS, LC and PFS rates were 94%, 89% and 81%, respectively. Despite the use of 3D-IGBT, larger tumors were significantly associated with poor LC. Therefore, other approaches, such as ISBT, may be more appropriate for improving the quality of brachytherapy plans associated with inadequate target coverage. As stated above, we performed template-based ISBT for bulky or irregularly shaped gynecological tumors. In a dosimetric study, Oike *et al.* reported that the combined intracavitary/interstitial approach could be a viable alternative to ISBT for such gynecological tumors [15]. According to their 5-year follow-up results, Ohno *et al.* [7] recently reported that 17% of patients who received CT-based IGBT required a combined intracavitary/interstitial approach. They reported excellent 5-year OS and LC rates of 86% and 94%, respectively, and no significant differences in 5-year LC among patients with tumors of ≤4, 4–6 and >6 cm in diameter. Therefore, a combined intracavitary/interstitial approach may be equally as effective as template-based ISBT.

In both Europe and the USA, a dose of >80 Gy is recommended in order to achieve a favorable LC [16, 17]. In contrast, an excellent LC was recently reported in a series of Japanese studies using delivery of a low HR-CTV D_90_ with a threshold dose of 60 Gy [7–9]. In Japan, the CS technique has been established as the standard approach for achieving a shorter overall treatment time and for reducing late morbidity rates. In these studies, use of the CS technique during EBRT is probably reasonable for Japanese patients, despite the lower total dose to the HR-CTV D_90_. In the current study, the mean HR-CTV D_90_ was 73.4 Gy, and favorable results were achieved using CS. However, the HR-CTV D_90_ was not significantly associated with LC, which may be due to the relatively small median tumor size (38 mm) in our patients compared with that of other Japanese studies. In other words, we had a low percentage of patients with large tumors, in which the HR-CTV D_90_ was relatively low and for whom LC may eventually not be possible. In addition, it was shown in phantom studies that the cumulative dose to the HR CTV D_90_, without CS, was clearly lower than the result of simple summing of the ICBT and ERBT doses incorporating CS. [18, 19]. Taken together, these studies suggest that further investigation is needed to evaluate the dose to the HR-CTV (using the CS technique) required for achievement of LC.

AD and large tumor sizes are well-known negative predictive factors for LC [20, 21]. However, the precise relationship of histology and tumor size to LC is unclear. Therefore, we investigated the effect of tumor size on local recurrence risk depending on the histology of the SCC and AD. The logistic regression curve indicated that the effect of size was greater for AD than for SCC in terms of LC, and a log-rank test indicated that bulky ADs had negative impacts on LC and on OS and PFS. Although these results are only hypothesis-generating, and further studies with a larger number of cases and longer follow-up are needed to confirm the hypothesis, it is possible that different treatment strategies, including concurrent use of platinum-based doublet chemotherapy [13] and carbon ion radiotherapy [22], might be necessary for bulky ADs.

The current study has several limitations. First, the study had a retrospective design using data from a single institution and with a limited follow-up period. Second, we did not examine combination brachytherapy with ICBT and ISBT, which offers scope for improvement in DVH parameters. Third, our treatment dose to the HR-CTV and the OARs during EBRT differed from that of most overseas studies because of the use of CS. Thus, it is difficult to compare the DVH parameters with those of other studies. Within these limitations, we found favorable outcomes and an acceptable incidence of late morbidity, comparable with previous studies. In patients with AD, the incidence of LC had a greater association with tumor size, compared with patients with SCC.
